# {(*S*)-2-[({2-[1-(Anthracen-9-ylmeth­yl)pyrrolidine-2-carboxamido]­phen­yl}(phen­yl)methyl­idene)amino]­acetato(2−)-κ^4^ *N*,*N*′,*N*′′,*O* ^1^}nickel(II)

**DOI:** 10.1107/S1600536812026827

**Published:** 2012-06-20

**Authors:** Zdeňka Padělková, Alexander Popkov, Milan Nádvorník

**Affiliations:** a Department of General and Inorganic Chemistry, Faculty of Chemical Technology, University of Pardubice, Studentská 573, 53210 Pardubice, Czech Republic; b Na Klínku 1082, 530 06 Pardubice, Czech Republic

## Abstract

The title compound, [Ni(C_35_H_29_N_3_O_3_)], includes a Schiff base ligand derived from (*S*)-1-[(anthracen-9-yl)meth­yl]-*N*-(2-benz­oyl­phen­yl)pyrrolidine-2-carboxamide and glycine. The Ni^II^ atom is coordinated by three N atoms [Ni—N = 1.937 (3), 1.850 (3) and 1.850 (3) Å] and one O atom [Ni—O = 1.859 (2) Å], resulting in a pseudo-square-planar coordination environment.

## Related literature

For preparation and evaluation of similar compounds in model reactions, see: Belokon *et al.* (1988 ▶); Kožíšek *et al.* (2004 ▶); Popkov *et al.* (2002 ▶, 2010 ▶). For an overview of application procedures, see: Popkov *et al.* (2005 ▶) and works cited therein. For NMR in solutions and similar highly unusual long-range spin–spin inter­actions, see: Jirman *et al.* (1998 ▶); Langer *et al.* (2007 ▶); Popkov *et al.* (1998 ▶, 2003 ▶). For the review of applications in positron emission tomography (PET), see: Popkov & De Spiegeleer (2012 ▶).
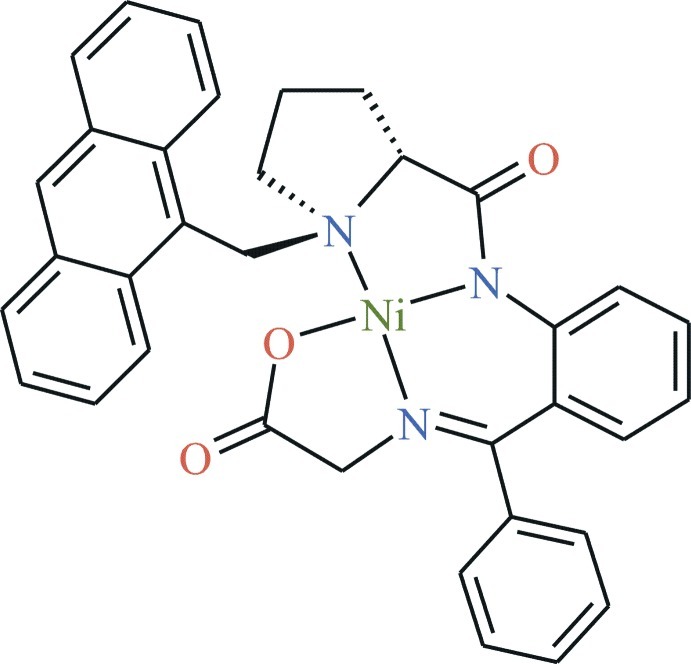


## Experimental

### 

#### Crystal data

[Ni(C_35_H_29_N_3_O_3_)]
*M* *_r_* = 598.32
Orthorhombic, 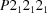

*a* = 8.9080 (5) Å
*b* = 16.5249 (12) Å
*c* = 18.6981 (13) Å
*V* = 2752.4 (3) Å^3^
*Z* = 4
Mo *K*α radiation
μ = 0.75 mm^−1^
*T* = 150 K
0.31 × 0.26 × 0.14 mm

#### Data collection

Bruker–Nonius KappaCCD area-detector diffractometer
Absorption correction: Gaussian (Coppens, 1970 ▶) *T*_min_ = 0.856, *T*_max_ = 0.925
23768 measured reflections
6120 independent reflections
5037 reflections with *I* > 2σ(*I*)
*R*_int_ = 0.071

#### Refinement

*R*[*F* ^2^ > 2σ(*F* ^2^)] = 0.044
*wR*(*F* ^2^) = 0.087
*S* = 1.19
6120 reflections
379 parameters
H-atom parameters constrained
Δρ_max_ = 0.32 e Å^−3^
Δρ_min_ = −0.38 e Å^−3^
Absolute structure: Flack (1983 ▶), 2615 Friedel pairs
Flack parameter: −0.019 (14)

### 

Data collection: *COLLECT* (Hooft, 1998 ▶) and *DENZO* (Otwin­owski & Minor, 1997 ▶); cell refinement: *COLLECT* and *DENZO*; data reduction: *COLLECT* and *DENZO*; program(s) used to solve structure: *SIR92* (Altomare *et al.*, 1994 ▶); program(s) used to refine structure: *SHELXL97* (Sheldrick, 2008 ▶); molecular graphics: *PLATON* (Spek, 2009 ▶); software used to prepare material for publication: *SHELXL97*.

## Supplementary Material

Crystal structure: contains datablock(s) I, global. DOI: 10.1107/S1600536812026827/im2378sup1.cif

Structure factors: contains datablock(s) I. DOI: 10.1107/S1600536812026827/im2378Isup2.hkl

Additional supplementary materials:  crystallographic information; 3D view; checkCIF report

## Figures and Tables

**Fig. 1.**
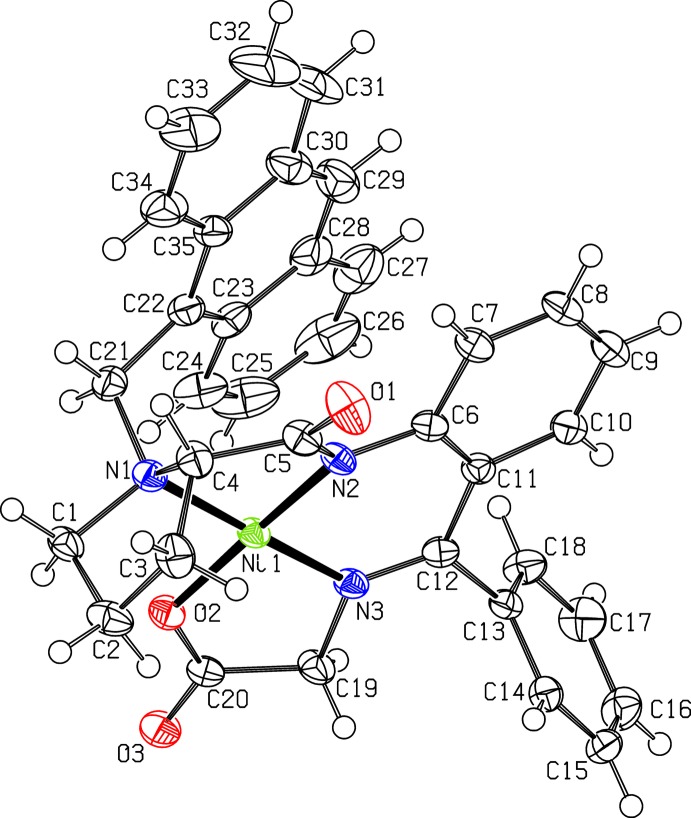
Molecular structure of the title compounds with displacement ellipsoids shown at the 50% probability level. H atoms are shown as spheres with arbitrary radii.

## supplementary crystallographic information

### Comment

Preparation of carbon-11 and fluorine-18 labelled amino acids for positron emission tomography (PET) is a big challenge for radiochemists. Due to time constrains brought by short half-life of the both isotopes, chromatographic separation steps should be avoided in PET radiosyntheses unless absolutely necessary (Popkov & De Spiegeleer (2012)). In order to meet this requirement we have been developing enantiospecific and highly enantioselective amino acid synthons based on Belokon's nickel(II) complexes (Belokon, *et al.*, 1988). We already demonstrated the origin of the high stereoselectivity of the incorporation of amino acid side chains into these synthons. Intramolecular electrostatic interaction of the (substituted) benzyl ring and the nickel atom (Kožíšek *et al.*, 2004) play a very important role as well as steric shielding by *ortho*-substituents of the benzyl ring (Popkov, *et al.*, 2002). In this communication we describe the crystal structure of the nickel(II) complex with an electron-rich (9-antracenyl)methyl substituent at the nitrogen atom of the proline residue due to the fact that the Schiff base ligand was derived from (*S*)-*N*-(2-benzoylphenyl)1-(9-antracenyl)methylpyrrolidine-2-carboxamide and glycine (AMGK). This structure is a candidate for charge density measurement. Recently, we have shown such complexes to be very efficient synthons of glycine or alanine for the preparation of radiotracers for PET (Popkov *et al.*, 2010). Similar complexes demonstrated highly unusual long-range spin-spin interactions in ^13^C-^13^C and ^15^N-^13^C NMR spectra (Jirman *et al.*, 1998; Popkov *et al.*, 1998; Langer *et al.*, 2007). These interactions have been attributed to the influence of a diffuse electron cloud from the benzyl group (Popkov *et al.*, 2003). We expect such interactions to be more pronounced in AMGK. For the future charge density measurement it is important that the conformation of AMGK described in this communication is similar to the conformation of the Ni^II^ complex of Schiff base of (*S*)-*N*-(2-benzoylphenyl)-1-benzylpyrrolidine-2-carboxamide and glycine (GK) (Popkov *et al.*, 2003) which is the simplest complex in this class and which was comprehensively studied by diffraction of X-rays and by NMR in solutions (Popkov *et al.*, 1998; Kožíšek *et al.*, 2004). In the solid state both complexes exhibit no intra- or intermolecular hydrogen bonds. The crystal packing is therefore only determined by weak interactions. Packing of the molecules in both crystals as well as the conformations are very similar, although the conformations of the molecules themselves differ. In the [Ni(GK)] complex intramolecular interactions are weaker as exemplified by the distance Ni-C22 (2.9282 (17) Å) and the angles Ni-N1-C21 (107.53 (9)°) and N1-C21-C22 (114.04 (13)°), respectively. In the complex [Ni(AMGK)] (Fig. 1) much stronger intramolecular interactions are observed shown by the distance Ni-C22 (3.181 (3) Å) and the angles Ni-N1-C21 (111.82 (19)°) and N1-C21-C22 (114.9 (2)°). Bulkiness of the anthranylmethyl group practically does not change the conformation of the molecule. The interatomic distance Ni-C22 in the more sterically hindered complex is just 0.253Å longer which is not too big difference compared to the published data for (substituted) analogues of GK (Popkov *et al.* (2003)). MP2 *ab initio* modelling of the interactions is in progress.

### Experimental

The title compound has been prepared according to a procedure described elsewhere (Popkov *et al.*, 2010). Crystals suitable for the measurement were obtained by slow evaporation of the solvent from a solution of the title compound in toluene/methanol (2:1).

### Refinement

Hydrogen atoms were mostly localized on a difference Fourier map, however to ensure uniformity of treatment of crystal, all hydrogen were recalculated into idealized positions (riding model) and assigned temperature factors U_iso_(H) = 1.2 U_eq_(pivot atom) or of 1.5 U_eq_ for the methyl moiety with C-H = 0.97, 0.98 and 0.93 Å for methylene, methine and hydrogen atoms at ain aromatic ring, respectively.

### Crystal data

**Table:**

[Ni(C_35_H_29_N_3_O_3_)]	*F*(000) = 1248
*M**_r_* = 598.32	*D*_x_ = 1.444 Mg m^−^^3^
Orthorhombic, *P*2_1_2_1_2_1_	Mo *K*α radiation, λ = 0.71073 Å
Hall symbol: P 2ac 2ab	Cell parameters from 23827 reflections
*a* = 8.9080 (5) Å	θ = 1–27.5°
*b* = 16.5249 (12) Å	µ = 0.75 mm^−^^1^
*c* = 18.6981 (13) Å	*T* = 150 K
*V* = 2752.4 (3) Å^3^	Block, red
*Z* = 4	0.31 × 0.26 × 0.14 mm

### Data collection

**Table:**

Bruker–Nonius KappaCCD area-detector diffractometer	6120 independent reflections
Radiation source: fine-focus sealed tube	5037 reflections with *I* > 2σ(*I*)
Graphite monochromator	*R*_int_ = 0.071
Detector resolution: 9.091 pixels mm^-1^	θ_max_ = 27.5°, θ_min_ = 1.6°
φ and ω scans to fill the Ewald sphere	*h* = −11→10
Absorption correction: gaussian (Coppens, 1970)	*k* = −19→21
*T*_min_ = 0.856, *T*_max_ = 0.925	*l* = −22→24
23768 measured reflections	

### Refinement

**Table:**

Refinement on *F*^2^	Secondary atom site location: difference Fourier map
Least-squares matrix: full	Hydrogen site location: inferred from neighbouring sites
*R*[*F*^2^ > 2σ(*F*^2^)] = 0.044	H-atom parameters constrained
*wR*(*F*^2^) = 0.087	*w* = 1/[σ^2^(*F*_o_^2^) + (0.0124*P*)^2^ + 2.2393*P*] where *P* = (*F*_o_^2^ + 2*F*_c_^2^)/3
*S* = 1.19	(Δ/σ)_max_ = 0.001
6120 reflections	Δρ_max_ = 0.32 e Å^−^^3^
379 parameters	Δρ_min_ = −0.38 e Å^−^^3^
0 restraints	Absolute structure: Flack (1983), 2615 Friedel pairs
Primary atom site location: structure-invariant direct methods	Flack parameter: −0.019 (14)

### Special details

**Table:**

Geometry. All esds (except the esd in the dihedral angle between two l.s. planes) are estimated using the full covariance matrix. The cell esds are taken into account individually in the estimation of esds in distances, angles and torsion angles; correlations between esds in cell parameters are only used when they are defined by crystal symmetry. An approximate (isotropic) treatment of cell esds is used for estimating esds involving l.s. planes.
Refinement. Refinement of F^2^ against ALL reflections. The weighted R-factor wR and goodness of fit S are based on F^2^, conventional R-factors R are based on F, with F set to zero for negative F^2^. The threshold expression of F^2^ > 2sigma(F^2^) is used only for calculating R-factors(gt) etc. and is not relevant to the choice of reflections for refinement. R-factors based on F^2^ are statistically about twice as large as those based on F, and R- factors based on ALL data will be even larger.

### Fractional atomic coordinates and isotropic or equivalent isotropic displacement parameters (Å^2^)

**Table:**

	*x*	*y*	*z*	*U*_iso_*/*U*_eq_	
Ni1	0.24182 (5)	0.29142 (2)	0.341302 (19)	0.01922 (9)	
O2	0.3360 (2)	0.30227 (14)	0.42952 (11)	0.0254 (5)	
N3	0.0779 (3)	0.34255 (16)	0.38193 (14)	0.0205 (6)	
N1	0.4180 (3)	0.24291 (16)	0.29833 (14)	0.0210 (6)	
N2	0.1482 (3)	0.27289 (15)	0.25450 (14)	0.0211 (6)	
O3	0.2972 (2)	0.34356 (15)	0.54199 (12)	0.0308 (6)	
O1	0.1528 (3)	0.17305 (14)	0.16634 (15)	0.0376 (6)	
C7	−0.0029 (4)	0.3172 (2)	0.15323 (19)	0.0258 (7)	
H7	0.0546	0.2854	0.1227	0.031*	
C14	−0.2558 (4)	0.36460 (18)	0.43374 (16)	0.0243 (6)	
H14	−0.2499	0.3085	0.4308	0.029*	
C5	0.2084 (3)	0.2090 (2)	0.21725 (17)	0.0246 (7)	
C20	0.2548 (4)	0.33210 (17)	0.48055 (15)	0.0226 (6)	
C18	−0.1673 (4)	0.4966 (2)	0.3992 (2)	0.0295 (8)	
H18	−0.1027	0.5289	0.3726	0.035*	
C12	−0.0396 (3)	0.37223 (18)	0.34982 (18)	0.0210 (7)	
C13	−0.1571 (4)	0.41254 (19)	0.39428 (17)	0.0210 (7)	
C6	0.0284 (4)	0.3172 (2)	0.22744 (18)	0.0216 (7)	
C21	0.5180 (3)	0.3056 (2)	0.26366 (17)	0.0249 (7)	
H21A	0.5682	0.3362	0.3009	0.030*	
H21B	0.5947	0.2777	0.2364	0.030*	
C4	0.3570 (4)	0.18159 (19)	0.24694 (18)	0.0248 (7)	
H4	0.4284	0.1733	0.2078	0.030*	
C22	0.4380 (4)	0.3641 (2)	0.21447 (18)	0.0258 (8)	
C2	0.4126 (4)	0.1213 (2)	0.36427 (19)	0.0315 (8)	
H2A	0.3359	0.1345	0.3992	0.038*	
H2B	0.4709	0.0757	0.3816	0.038*	
C15	−0.3628 (4)	0.4004 (2)	0.47780 (19)	0.0300 (8)	
H15	−0.4290	0.3684	0.5039	0.036*	
C11	−0.0600 (4)	0.36713 (19)	0.27272 (17)	0.0210 (7)	
C9	−0.2021 (4)	0.4135 (2)	0.16876 (19)	0.0297 (8)	
H9	−0.2780	0.4452	0.1493	0.036*	
C1	0.5114 (3)	0.1935 (2)	0.34910 (18)	0.0258 (7)	
H1A	0.5331	0.2235	0.3925	0.031*	
H1B	0.6051	0.1771	0.3270	0.031*	
C16	−0.3702 (4)	0.4840 (2)	0.4825 (2)	0.0352 (9)	
H16	−0.4409	0.5080	0.5123	0.042*	
C8	−0.1158 (4)	0.3641 (2)	0.12543 (19)	0.0291 (8)	
H8	−0.1348	0.3628	0.0765	0.035*	
C30	0.3486 (5)	0.4049 (2)	0.0956 (2)	0.0354 (9)	
C10	−0.1745 (4)	0.4141 (2)	0.24071 (19)	0.0262 (7)	
H10	−0.2332	0.4471	0.2697	0.031*	
C24	0.3850 (5)	0.4572 (2)	0.3174 (2)	0.0379 (10)	
H24	0.4428	0.4251	0.3476	0.045*	
C35	0.4320 (4)	0.3507 (2)	0.13999 (18)	0.0276 (8)	
C19	0.0938 (4)	0.3502 (2)	0.45967 (17)	0.0264 (8)	
H19A	0.0265	0.3128	0.4835	0.032*	
H19B	0.0678	0.4047	0.4743	0.032*	
C34	0.5118 (4)	0.2865 (3)	0.10405 (19)	0.0364 (8)	
H34	0.5675	0.2500	0.1310	0.044*	
C28	0.2807 (4)	0.4858 (2)	0.1981 (2)	0.0341 (9)	
C23	0.3690 (4)	0.4337 (2)	0.2440 (2)	0.0283 (8)	
C3	0.3427 (4)	0.1027 (2)	0.2911 (2)	0.0319 (8)	
H3A	0.2380	0.0877	0.2963	0.038*	
H3B	0.3956	0.0586	0.2679	0.038*	
C17	−0.2732 (5)	0.5318 (2)	0.4430 (2)	0.0355 (9)	
H17	−0.2786	0.5878	0.4464	0.043*	
C25	0.3180 (5)	0.5244 (2)	0.3438 (3)	0.0540 (12)	
H25	0.3341	0.5394	0.3911	0.065*	
C33	0.5074 (5)	0.2784 (3)	0.0321 (2)	0.0535 (12)	
H33	0.5601	0.2363	0.0107	0.064*	
C31	0.3485 (6)	0.3928 (3)	0.0199 (2)	0.0557 (13)	
H31	0.2938	0.4279	−0.0089	0.067*	
C29	0.2727 (5)	0.4695 (2)	0.1259 (2)	0.0407 (10)	
H29	0.2140	0.5025	0.0969	0.049*	
C26	0.2233 (6)	0.5723 (3)	0.2992 (3)	0.0592 (15)	
H26	0.1728	0.6165	0.3183	0.071*	
C27	0.2067 (4)	0.5540 (2)	0.2292 (3)	0.0501 (12)	
H27	0.1455	0.5864	0.2009	0.060*	
C32	0.4254 (6)	0.3324 (3)	−0.0107 (2)	0.0650 (15)	
H32	0.4247	0.3262	−0.0601	0.078*	

### Atomic displacement parameters (Å^2^)

**Table:**

	*U*^11^	*U*^22^	*U*^33^	*U*^12^	*U*^13^	*U*^23^
Ni1	0.02184 (17)	0.02218 (17)	0.01363 (16)	0.0019 (2)	−0.00030 (18)	−0.00127 (17)
O2	0.0269 (11)	0.0340 (14)	0.0153 (11)	0.0000 (11)	−0.0023 (9)	−0.0030 (11)
N3	0.0244 (14)	0.0227 (14)	0.0145 (14)	0.0016 (11)	0.0007 (11)	−0.0004 (11)
N1	0.0241 (14)	0.0218 (14)	0.0170 (14)	0.0009 (11)	0.0001 (11)	−0.0008 (11)
N2	0.0273 (14)	0.0218 (15)	0.0144 (13)	0.0023 (11)	−0.0034 (11)	−0.0012 (11)
O3	0.0336 (14)	0.0419 (15)	0.0170 (12)	−0.0017 (11)	−0.0014 (9)	−0.0034 (11)
O1	0.0482 (14)	0.0312 (13)	0.0334 (15)	0.0079 (11)	−0.0156 (13)	−0.0145 (12)
C7	0.0321 (17)	0.0277 (18)	0.0176 (17)	0.0001 (14)	−0.0030 (15)	−0.0016 (15)
C14	0.0232 (15)	0.0272 (15)	0.0225 (15)	−0.0020 (17)	−0.0029 (16)	0.0003 (12)
C5	0.0318 (18)	0.0224 (15)	0.0197 (15)	0.0027 (15)	−0.0005 (12)	−0.0036 (15)
C20	0.0284 (15)	0.0231 (14)	0.0162 (14)	0.0000 (17)	0.0001 (17)	0.0004 (11)
C18	0.0340 (19)	0.0246 (18)	0.030 (2)	0.0003 (16)	0.0079 (16)	0.0028 (16)
C12	0.0255 (15)	0.0153 (15)	0.0220 (18)	−0.0023 (13)	0.0019 (13)	0.0014 (14)
C13	0.0231 (16)	0.0236 (17)	0.0162 (16)	0.0008 (14)	−0.0005 (13)	0.0021 (13)
C6	0.0266 (17)	0.0208 (17)	0.0173 (17)	−0.0006 (13)	0.0010 (13)	−0.0006 (13)
C21	0.0230 (16)	0.032 (2)	0.0196 (17)	−0.0019 (15)	0.0011 (12)	−0.0032 (15)
C4	0.0278 (17)	0.0281 (18)	0.0184 (17)	0.0051 (14)	−0.0005 (14)	−0.0070 (14)
C22	0.0250 (17)	0.030 (2)	0.0228 (18)	−0.0053 (15)	0.0019 (14)	0.0034 (15)
C2	0.045 (2)	0.0235 (19)	0.026 (2)	0.0072 (16)	0.0020 (15)	0.0050 (14)
C15	0.0225 (17)	0.045 (2)	0.0225 (19)	−0.0059 (16)	0.0019 (14)	0.0027 (16)
C11	0.0227 (16)	0.0197 (17)	0.0206 (17)	−0.0029 (13)	−0.0010 (13)	0.0024 (13)
C9	0.0333 (18)	0.0305 (18)	0.025 (2)	0.0022 (14)	−0.0065 (14)	0.0060 (16)
C1	0.0287 (15)	0.0320 (19)	0.0165 (16)	0.0092 (14)	−0.0006 (13)	−0.0007 (15)
C16	0.0296 (19)	0.048 (2)	0.028 (2)	0.0051 (17)	0.0049 (16)	−0.0073 (18)
C8	0.037 (2)	0.032 (2)	0.0185 (17)	0.0005 (16)	−0.0059 (14)	0.0037 (15)
C30	0.044 (2)	0.032 (2)	0.029 (2)	−0.0119 (18)	−0.0077 (17)	0.0065 (17)
C10	0.0266 (17)	0.0279 (18)	0.0241 (19)	0.0023 (14)	0.0015 (14)	0.0037 (15)
C24	0.051 (2)	0.031 (2)	0.032 (2)	−0.0039 (18)	0.0139 (18)	−0.0006 (17)
C35	0.0278 (17)	0.035 (2)	0.0197 (19)	−0.0051 (15)	0.0012 (13)	0.0035 (14)
C19	0.0296 (18)	0.034 (2)	0.0156 (17)	0.0082 (15)	0.0003 (13)	−0.0015 (14)
C34	0.042 (2)	0.045 (2)	0.0228 (19)	−0.004 (2)	0.0042 (15)	−0.0018 (19)
C28	0.030 (2)	0.0249 (18)	0.047 (2)	−0.0092 (15)	0.0032 (17)	0.0067 (16)
C23	0.0252 (17)	0.0260 (18)	0.034 (2)	−0.0071 (15)	0.0066 (15)	0.0019 (16)
C3	0.033 (2)	0.0241 (18)	0.039 (2)	0.0053 (16)	−0.0028 (16)	−0.0011 (16)
C17	0.043 (2)	0.0275 (17)	0.037 (2)	0.0073 (18)	0.0062 (18)	−0.0041 (15)
C25	0.080 (3)	0.038 (2)	0.043 (3)	−0.004 (2)	0.029 (3)	−0.006 (2)
C33	0.070 (3)	0.062 (3)	0.028 (2)	−0.003 (3)	0.010 (2)	−0.008 (2)
C31	0.080 (3)	0.061 (3)	0.025 (2)	−0.013 (3)	−0.018 (2)	0.015 (2)
C29	0.042 (2)	0.035 (2)	0.046 (2)	−0.008 (2)	−0.013 (2)	0.0141 (17)
C26	0.067 (4)	0.033 (2)	0.077 (4)	0.005 (2)	0.039 (3)	−0.002 (2)
C27	0.041 (3)	0.033 (2)	0.076 (4)	−0.0018 (18)	0.014 (2)	0.013 (2)
C32	0.103 (4)	0.076 (4)	0.015 (2)	−0.019 (3)	−0.008 (2)	0.001 (2)

### Geometric parameters (Å, º)

**Table:**

Ni1—N2	1.850 (3)	C15—H15	0.9300
Ni1—N3	1.850 (3)	C11—C10	1.415 (4)
Ni1—O2	1.859 (2)	C9—C10	1.368 (5)
Ni1—N1	1.937 (3)	C9—C8	1.383 (5)
O2—C20	1.295 (4)	C9—H9	0.9300
N3—C12	1.303 (4)	C1—H1A	0.9701
N3—C19	1.466 (4)	C1—H1B	0.9700
N1—C4	1.499 (4)	C16—C17	1.384 (5)
N1—C1	1.503 (4)	C16—H16	0.9300
N1—C21	1.512 (4)	C8—H8	0.9300
N2—C5	1.374 (4)	C30—C29	1.385 (6)
N2—C6	1.389 (4)	C30—C35	1.429 (5)
O3—C20	1.224 (4)	C30—C31	1.431 (6)
O1—C5	1.226 (4)	C10—H10	0.9300
C7—C8	1.372 (5)	C24—C25	1.354 (5)
C7—C6	1.415 (5)	C24—C23	1.433 (5)
C7—H7	0.9300	C24—H24	0.9299
C14—C15	1.392 (5)	C35—C34	1.442 (5)
C14—C13	1.395 (4)	C19—H19A	0.9701
C14—H14	0.9299	C19—H19B	0.9701
C5—C4	1.505 (4)	C34—C33	1.353 (5)
C20—C19	1.516 (5)	C34—H34	0.9301
C18—C17	1.378 (5)	C28—C29	1.379 (5)
C18—C13	1.394 (5)	C28—C27	1.429 (6)
C18—H18	0.9300	C28—C23	1.447 (5)
C12—C11	1.455 (4)	C3—H3A	0.9701
C12—C13	1.493 (4)	C3—H3B	0.9700
C6—C11	1.421 (4)	C17—H17	0.9299
C21—C22	1.513 (5)	C25—C26	1.426 (7)
C21—H21A	0.9700	C25—H25	0.9300
C21—H21B	0.9700	C33—C32	1.403 (7)
C4—C3	1.548 (5)	C33—H33	0.9300
C4—H4	0.9800	C31—C32	1.339 (7)
C22—C35	1.411 (5)	C31—H31	0.9300
C22—C23	1.417 (5)	C29—H29	0.9300
C2—C1	1.511 (5)	C26—C27	1.350 (7)
C2—C3	1.534 (5)	C26—H26	0.9300
C2—H2A	0.9701	C27—H27	0.9300
C2—H2B	0.9701	C32—H32	0.9300
C15—C16	1.386 (5)		
			
N2—Ni1—N3	94.59 (12)	C10—C9—H9	120.7
N2—Ni1—O2	176.00 (11)	C8—C9—H9	120.5
N3—Ni1—O2	87.01 (11)	N1—C1—C2	103.0 (3)
N2—Ni1—N1	86.14 (11)	N1—C1—H1A	111.2
N3—Ni1—N1	177.26 (12)	C2—C1—H1A	111.3
O2—Ni1—N1	92.43 (11)	N1—C1—H1B	111.1
C20—O2—Ni1	116.0 (2)	C2—C1—H1B	111.1
C12—N3—C19	120.2 (3)	H1A—C1—H1B	109.2
C12—N3—Ni1	128.1 (2)	C17—C16—C15	120.4 (3)
C19—N3—Ni1	111.7 (2)	C17—C16—H16	119.9
C4—N1—C1	103.8 (2)	C15—C16—H16	119.8
C4—N1—C21	113.7 (3)	C7—C8—C9	121.2 (3)
C1—N1—C21	108.4 (2)	C7—C8—H8	119.5
C4—N1—Ni1	104.59 (19)	C9—C8—H8	119.2
C1—N1—Ni1	114.3 (2)	C29—C30—C35	120.0 (3)
C21—N1—Ni1	111.8 (2)	C29—C30—C31	120.8 (4)
C5—N2—C6	121.3 (3)	C35—C30—C31	119.2 (4)
C5—N2—Ni1	113.3 (2)	C9—C10—C11	122.8 (3)
C6—N2—Ni1	125.3 (2)	C9—C10—H10	118.5
C8—C7—C6	121.1 (3)	C11—C10—H10	118.7
C8—C7—H7	119.4	C25—C24—C23	121.8 (4)
C6—C7—H7	119.6	C25—C24—H24	119.4
C15—C14—C13	120.2 (3)	C23—C24—H24	118.8
C15—C14—H14	119.8	C22—C35—C30	119.6 (3)
C13—C14—H14	120.0	C22—C35—C34	123.8 (3)
O1—C5—N2	127.5 (3)	C30—C35—C34	116.5 (3)
O1—C5—C4	119.7 (3)	N3—C19—C20	109.3 (3)
N2—C5—C4	112.8 (3)	N3—C19—H19A	110.0
O3—C20—O2	125.3 (3)	C20—C19—H19A	109.9
O3—C20—C19	120.2 (3)	N3—C19—H19B	109.7
O2—C20—C19	114.4 (3)	C20—C19—H19B	109.6
C17—C18—C13	120.3 (3)	H19A—C19—H19B	108.3
C17—C18—H18	119.9	C33—C34—C35	121.5 (4)
C13—C18—H18	119.8	C33—C34—H34	119.4
N3—C12—C11	122.3 (3)	C35—C34—H34	119.2
N3—C12—C13	118.3 (3)	C29—C28—C27	121.9 (4)
C11—C12—C13	119.3 (3)	C29—C28—C23	119.6 (4)
C18—C13—C14	119.3 (3)	C27—C28—C23	118.5 (4)
C18—C13—C12	121.8 (3)	C22—C23—C24	123.3 (3)
C14—C13—C12	118.9 (3)	C22—C23—C28	119.2 (3)
N2—C6—C7	120.6 (3)	C24—C23—C28	117.5 (3)
N2—C6—C11	121.0 (3)	C2—C3—C4	105.9 (3)
C7—C6—C11	118.3 (3)	C2—C3—H3A	110.6
N1—C21—C22	114.9 (3)	C4—C3—H3A	110.4
N1—C21—H21A	108.8	C2—C3—H3B	110.5
C22—C21—H21A	108.6	C4—C3—H3B	110.7
N1—C21—H21B	108.4	H3A—C3—H3B	108.7
C22—C21—H21B	108.4	C18—C17—C16	120.2 (3)
H21A—C21—H21B	107.5	C18—C17—H17	119.7
N1—C4—C5	110.6 (3)	C16—C17—H17	120.0
N1—C4—C3	104.9 (3)	C24—C25—C26	120.1 (5)
C5—C4—C3	112.2 (3)	C24—C25—H25	119.8
N1—C4—H4	109.7	C26—C25—H25	120.0
C5—C4—H4	109.7	C34—C33—C32	121.3 (5)
C3—C4—H4	109.6	C34—C33—H33	119.2
C35—C22—C23	119.7 (3)	C32—C33—H33	119.5
C35—C22—C21	121.2 (3)	C32—C31—C30	121.8 (4)
C23—C22—C21	119.1 (3)	C32—C31—H31	119.1
C1—C2—C3	103.1 (3)	C30—C31—H31	119.1
C1—C2—H2A	111.0	C28—C29—C30	121.7 (4)
C3—C2—H2A	111.1	C28—C29—H29	119.1
C1—C2—H2B	111.3	C30—C29—H29	119.3
C3—C2—H2B	111.1	C27—C26—C25	120.5 (4)
H2A—C2—H2B	109.1	C27—C26—H26	119.7
C16—C15—C14	119.6 (3)	C25—C26—H26	119.8
C16—C15—H15	120.2	C26—C27—C28	121.4 (4)
C14—C15—H15	120.2	C26—C27—H27	119.2
C10—C11—C6	117.8 (3)	C28—C27—H27	119.5
C10—C11—C12	118.5 (3)	C31—C32—C33	119.8 (4)
C6—C11—C12	123.7 (3)	C31—C32—H32	120.1
C10—C9—C8	118.8 (3)	C33—C32—H32	120.1
